# Implementation of national guidelines on antenatal magnesium sulfate for neonatal neuroprotection: extended evaluation of the effectiveness and cost-effectiveness of the National PReCePT Programme in England

**DOI:** 10.1136/bmjqs-2024-017763

**Published:** 2025-04-27

**Authors:** Hannah B Edwards, Carlos Sillero-Rejon, Hugh McLeod, Elizabeth M Hill, Brent C Opmeer, Colin Peters, David Odd, Frank de Vocht, Karen Luyt

**Affiliations:** 1 Population Health Sciences, Bristol Medical School, University of Bristol, Bristol, UK; 2 NIHR ARC West, Bristol, UK; 3 Vilans National Centre of Expertise in Long Term Care, Utrecht, The Netherlands; 4 Neonatology, Royal Hospital for Children, Glasgow, UK; 5 Population Medicine, Cardiff University, Cardiff, UK; 6 Neonatology, Cardiff and Vale University Health Board, Cardiff, UK; 7 Translational Health Sciences, University of Bristol Medical School, Bristol, Avon, UK; 8 Tertiary NICU, University Hospitals Bristol and Weston NHS Foundation Trust, Bristol, Avon, UK

**Keywords:** Cost-effectiveness, Obstetrics and gynecology, Evidence-based medicine, Healthcare quality improvement, Health services research

## Abstract

**Background:**

Since 2015, the National Institute for Health and Care Excellence (NICE) guidelines have recommended antenatal magnesium sulfate (MgSO_4_) for mothers in preterm labour (<30 weeks’ gestation) to reduce the risk of cerebral palsy (CP) in the preterm baby. However, the implementation of this guideline in clinical practice was slow, and MgSO_4_ use varied between maternity units. In 2018, the PRrevention of Cerebral palsy in PreTerm labour (PReCePT) programme, an evidence-based quality improvement (QI) intervention to improve use of MgSO_4_, was rolled out across England. Earlier evaluation found this programme to be effective and cost-effective over the first 12 months. We extended the original evaluation to determine the programme’s longer-term impact over 4 years, its impact in later preterm births, the impact of the COVID-19 pandemic, and to compare MgSO_4_ use in England (where PReCePT was implemented) to Scotland and Wales (where it was not).

**Methods:**

Quasi-experimental longitudinal study using data from the National Neonatal Research Database on babies born <30 weeks’ gestation and admitted to a National Health Service neonatal unit. Primary outcome was the percentage of eligible mothers receiving MgSO_4_, aggregated to the national level. Impact of PReCePT on MgSO_4_ use was estimated using multivariable linear regression. The net monetary benefit (NMB) of the programme was estimated.

**Results:**

MgSO_4_ administration rose from 65.8% in 2017 to 85.5% in 2022 in England. PReCePT was associated with a 5.8 percentage points improvement in uptake (95% CI 2.69 to 8.86, p<0.001). Improvement was greater when including older preterm births (<34 weeks’ gestation, 8.67 percentage points, 95% CI 6.38 to 10.96, p<0.001). Most gains occurred in the first 2 years following implementation. PReCePT had a NMB of £597 000 with 89% probability of being cost-effective. Following implementation, English uptake appeared to accelerate compared with Scotland and Wales. There was some decline in use coinciding with the onset of the pandemic.

**Conclusions:**

The PReCePT QI programme cost-effectively improved use of antenatal MgSO_4_, with anticipated benefits to the babies who have been protected from CP.

**WHAT IS ALREADY KNOWN ON THIS TOPIC**

- Antenatal magnesium sulfate (MgSO_4_) reduces the risk of cerebral palsy in babies born preterm.
- The National PRevention of Cerebral palsy in PreTerm labour (PReCePT) Quality Improvement (QI) Programme (NPP) effectively and cost-effectively improved use of MgSO_4_ in England in the first 12 months of implementation, but sustaining QIs over time is often challenging.

**WHAT THIS STUDY ADDS**

- Using a quasi-experimental design and routinely collected, longitudinal, patient-level data, this study found that the NPP had sustained effectiveness and cost-effectiveness over 4 years following implementation.
- Improvement may have been accelerated in England, compared with Scotland and Wales, where the NPP was not formally implemented.

**HOW THIS STUDY MIGHT AFFECT RESEARCH, PRACTICE OR POLICY**

- This study demonstrates that dedicated national programmes can cost-effectively achieve improvements in perinatal care; the PReCePT model could be used as an implementation blueprint for other QI initiatives in perinatal care.

## Introduction

 Since 2015, the WHO^1^ and the National Institute for Health and Care Excellence (NICE)^2^ guidelines (which officially apply to England and partly or more flexibly in Wales and Scotland) have recommended administration of magnesium sulfate (MgSO_4_) in preterm deliveries <30 weeks’ gestation as a core part of maternity care. This follows strong evidence that when given antenatally to women in preterm labour, MgSO_4_ reduces the risk of cerebral palsy (CP) in preterm babies by around 30%.^3^ Historically, use of this treatment has been inconsistent, with only 64% of eligible women in England being treated in 2017. High regional variation in uptake also indicates inequalities in perinatal care.^4^

As well as the significant impact of CP on affected individuals and their families,^5^ there are lifetime societal costs of approximately £1 m per affected individual^6^ and £1.8 billion annually on National Health Service (NHS) clinical negligence litigation (half of the total NHS litigation expenditure).^7^ Incidence of CP has been estimated at around 1.5 per 1000 livebirths in the UK,^8^ with preterm birth as the leading risk factor.^9^,^11^ This highlights the importance of funding effective and cost-effective strategies to reduce the risk of CP associated with preterm birth. It is estimated that one case of CP can be prevented for every 37 mothers (<30 weeks' gestation) treated with MgSO_4_, and around 200 cases of CP per year could be avoided by consistent administration of MgSO_4_ during labour.^3^

In 2018, NHS England rolled out the National PReCePT (PRevention of Cerebral palsy in PreTerm labour) Programme (NPP). This was a quality improvement (QI) programme for maternity units, providing clinical guidance, training, learning resources, midwife backfill funding, and QI support, to improve maternity staff awareness and increase use of MgSO_4_ for mothers in preterm labour. The aim was to reach ≥85% uptake in eligible mothers (those expected to deliver <30 weeks' gestational age) across all maternity units in England. The programme was delivered by regional Academic Health Science Networks (AHSNs, now Health Innovation Networks). Evaluation of the first 12 months of the programme found it to be effective, improving MgSO_4_ use by an estimated 6.3 percentage points (95% CI 2.6 to 10.0 percentage points). Overall uptake rose from an average of 70.9% across the 12 months preintervention to 83.1% across the 12 months postintervention. This increase in MgSO_4_ uptake, when assessed in light of NPP implementation costs and lifetime societal costs of CP, was associated with an estimated net monetary benefit (NMB) of £866 per preterm baby (>95% probability of being cost-effective).^12^ However, it is unknown whether the improvement in MgSO_4_ uptake was sustained over time, and sustainability in large-scale implementation programmes is often a problem.^13^

The primary aim of this study was to evaluate the NPP’s longer-term, sustained effectiveness and cost-effectiveness over the first 4 years following implementation. Secondary aims were to: explore the impact on all babies born up to 34 weeks’ gestation (NICE guidelines recommend treatment for births up to 30 weeks’ and ‘consideration of treatment’ for those up to 34 weeks’); explore the impact of the COVID-19 pandemic on MgSO_4_ use; and to compare MgSO_4_ use in England with that in the devolved nations of Scotland and Wales (who did not have the NPP, but have been implementing their own MgSO_4_ initiatives, eg, Maternity and Children Quality Improvement Collaborative (MCQIC) Preterm Perinatal Wellbeing Package^14^ and PERIPrem (Perinatal Excellence to Reduce Injury in Premature Birth) Cymru^15^). A qualitative workstream alongside this study explored how the devolved nations were responding to the NICE guidance, and is reported elsewhere.^16^

## Methods

### Design

This was a quasi-experimental study to evaluate the NPP’s effectiveness and cost-effectiveness. The preregistered statistical analysis plan and health economic analysis plan were uploaded to the Open Science Framework prior to analyses.

### Intervention

The intervention being implemented was the NPP, as described above and fully detailed elsewhere,^12^ to improve use of antenatal MgSO_4_ in preterm births.

### Setting

The setting was NHS maternity units in England, Scotland and Wales. Within maternity units, analysis was performed on aggregated data on babies born preterm <30 weeks’ gestation and admitted to an NHS neonatal unit, between January 2014 and December 2022. All maternity units in England, Scotland and Wales were included, excepting the five units in England that took part in the original PReCePT pilot study^17^ and were therefore not part of the NPP.

### Data sources

Data on eligible babies and their mothers were obtained from the National Neonatal Research Database, which holds individual-level, pseudonymised, routinely collected patient data on babies admitted to an NHS neonatal unit. These data were linked (on unit code) to NPP data on unit start dates. Costs associated with the NPP were estimated in the original evaluation.^12^

### Effectiveness evaluation

#### Outcome

The main implementation outcome was MgSO_4_ uptake over time. MgSO_4_ uptake was defined as the percentage of eligible mothers recorded as receiving MgSO_4_ at a maternity unit (computed per month). We followed the convention of nationally reported audit data, in that mothers with missing MgSO_4_ data were excluded from the calculation of MgSO_4_ uptake, and only data on singletons and the first born (ie, one infant) from each multiple birth were included in the calculation.

#### Descriptive analysis

Maternity unit and population characteristics, and MgSO_4_ use, were descriptively reported by nation and time period.

#### Primary analysis

Primary analysis was an interrupted time-series using English data aggregated to the national level (mean national MgSO_4_ uptake per month, across all English maternity units). A multivariable linear regression model was used to estimate the difference in mean MgSO_4_ uptake from before (the 1-year period before) to after (the 4 years follow-up) implementation of the NPP in England. The model adjusted for an underlying linear time trend, and mother and baby characteristics aggregated nationally per month (mean maternal age, Index of Multiple Deprivation decile,^18^ baby’s birth weight adjusted for gestational age as a z-score, and proportion reported smokers, white British ethnicity, type of birth (c-section vs vaginal delivery) and multiple births). The model was further adjusted for a non-linear temporal component to account for the ceiling effect at 100% uptake and reduction in the rate of change at levels close to the ceiling (improvement is not perfectly linear, tending to be faster when overall uptake is low, where ‘easy gains’ can be made). This was done via an interaction term between study month and NPP period. Data on paternal age and ethnicity were explored as potential confounding factors but were excluded due to high levels of missing data and expected collinearity with other variables (maternal and paternal age tends to correlate, as does maternal and paternal ethnicity). Potential interaction was explored between mean MgSO_4_ uptake and level of unit (neonatal intensive care unit (NICU), the highest-level unit, vs special care baby unit (SCBU) or local neonatal unit (LNU), lower-level units). This was because it was anticipated that performance might differ by type of unit: data from the original study indicated that lower-level units tended to have lower starting uptake levels, so more room for improvement compared with the higher-level NICUs.

#### Sensitivity and subgroup analyses

As sensitivity analyses, the above model was run on data aggregated to (1) the maternity unit level rather than the national level and (2) the individual rather than national level. These two models additionally adjusted for type of unit (NICU vs SCBU/LNU), regional clustering by AHSN, and were weighted on the number of eligible births per unit per month. Other sensitivity analyses included assessing (3) the impact of excluding a ‘fuzzy’ implementation start window of +/−2 months, to account for some units starting slightly earlier or later than their officially recorded start date; (4) the impact of excluding the final 2 months of data, due to concerns about completeness of the most recent data for some units; (5) the impact of using a longer pre-NPP comparison period of 4 years; and (6) the impact of including more mature preterm babies up to 34 weeks’ gestation in the analysis. A subgroup analysis was performed on the 40 units that had participated in a connected study, a randomised controlled trial (RCT) nested within the main NPP,^19^ as their performance could plausibly differ from other maternity units.

### Economic evaluation

#### MgSO_4_ treatment cost-effectiveness

Economic analysis combines evidence of the treatment (MgSO_4_) effect with evidence of the implementation (NPP) effect.^20^ For the former, estimates of the cost-effectiveness of MgSO_4_ treatment were adopted from Bickford and colleagues’ results,^6 21 22^ with their estimates converted to GBP (British Pound Sterling) currency and 2019 prices (online supplemental table 1). In summary, Bickford and colleagues conducted a decision analytical model to estimate the cost-effectiveness of MgSO_4_ in the prevention of CP in preterm births (<32 weeks’ gestation) including a lifetime and societal perspective. Their analysis shows that MgSO_4_ is a dominant strategy (ie, cost-effective), also observed in other evidence and NICE guidelines.^2 22 23^

#### NPP implementation costs and effectiveness

The total implementation cost of the NPP of £936 747 was estimated from data supplied by the NPP team and PReCePT study team, and reported previously.^12 19^ This represents a mean implementation cost per unit of £6044 per unit: £738 for NPP management, £2764 for AHSN regional support, and £2500 for clinical backfill-funding of clinical time for NPP ‘champion’ midwives at each unit.^12^

From the multivariable linear regression model described above, we estimated the NPP effectiveness as the difference between the estimated level of MgSO_4_ use over time compared with a counterfactual level of MgSO_4_ use, representing what may have occurred in the absence of the NPP, assuming a continuation of the pre-NPP trend in MgSO_4_ uptake (counterfactual was calculated monthly assuming the intervention did not occur—ie, zero, and, therefore, based only on time (month) and other covariates). The main measure of NPP effectiveness was the area between the curves. Primary analysis used linear regression to estimate the counterfactual based on the pre-NPP predicted trend. Sensitivity analysis used a beta regression (ie, uptake assumed to follow a beta distribution) to estimate this counterfactual, to account for MgSO_4_ uptake as a proportion with a ceiling effect at 100% uptake.

#### Policy cost-effectiveness analysis

The cost-effectiveness analysis was conducted from a societal lifetime perspective. NMB of the NPP was estimated over the 4 years since its launch, by combining analysis of the costs and effectiveness of the NPP with the lifetime societal cost and health gains associated with MgSO_4_ treatment. This analysis used a framework previously developed to conduct economic evaluations of implementation initiatives^24^ which is based on the methods of policy cost-effectiveness and value of implementation.^25 26^ The NMB of a treatment combines incremental costs and benefits into a single summary monetary statistic, using a willingness-to-pay (WTP) threshold for valuing quality-of-life gains—the amount that could be paid to achieve one additional quality-adjusted life year (QALY) and be viewed as representing value for money. If the NMB is positive, the intervention is cost-effective at the chosen WTP threshold. We used a WTP threshold of £20 000 per QALY gained, following NICE guidelines.^27^ Our framework extends the calculation of the treatment NMB by incorporating the impact of the implementation initiative in terms of additional patients treated (ie, uptake attributed to NPP) and the implementation costs (ie, for NPP). Similarly, a positive NMB value would indicate that the implementation initiative was cost-effective. The net increment in the number of patients that received MgSO_4_ and the implementation cost-effectiveness per additional patient treated were also estimated. The analysis used the area-between-the-curves estimate of NPP effectiveness with a linear counterfactual and a sensitivity analysis using a beta counterfactual.

Probabilistic analysis was conducted using a Monte Carlo simulation with 10 000 samples drawn from parameter distributions. Point estimates, probabilistic distribution assumptions and parameter source estimates are reported in online supplemental table 2. Cost-effectiveness planes and cost-effectiveness acceptability curves were plotted for WTP thresholds from 0 to £100 000 per QALY gained for the policy cost-effectiveness of the NPP intervention.

#### Secondary economic analysis

As evidence on the lifetime cost-effectiveness of antenatal MgSO_4_ covers babies born up to 32 weeks’ gestation, cost-effectiveness analysis was performed only for babies<30 and <32 weeks’ gestation. Older babies were not included in the economic analysis due to the lack of cost-effectiveness evidence on them.

Statistical software Stata V.17 and R V.4.3.1 (economic evaluation) were used for all statistical analyses.

## Results

### Baseline characteristics

In 2017, the year before NPP roll-out, a total of 4091 babies born under 30 weeks’ gestational age were admitted to neonatal units in England, 296 in Scotland and 182 in Wales. (For context, in 2017 the Office for National Statistics reported a total of 646 794 live births in England, 52 861 in Scotland and 32 176 in Wales^28^). The majority of births were in maternity units with an NICU (62.6% in England, 83.5% in Scotland, 63.0% in Wales). Other than the number of babies admitted, study populations were largely comparable across the three nations with respect to other covariates (table 1).

**Table 1:** Baby, mother and maternity unit characteristics by nation at baseline*

	England	Scotland	Wales
**Socio-demographic characteristics of babies**			
Number of babies (N)†	4091	296	182
Gestational age (median weeks, IQR)	27.7 (26.0–28.9)	27.9 (26.6–28.9)	28.0 (26.6–28.9)
22 weeks	14 (0.3%)	0 (0.0%)	3 (1.7%)
23 weeks	225 (5.5%)	12 (4.1%)	6 (3.3%)
24 weeks	374 (9.1%)	24 (8.5%)	9 (5.0%)
25 weeks	407 (10.0%)	19 (6.4%)	12 (6.6%)
26 weeks	530 (13.0%)	34 (11.5%)	22 (12.1%)
27 weeks	644 (15.7%)	58 (19.6%)	34 (18.7%)
28 weeks	886 (21.7%)	74 (25.0%)	56 (30.8%)
29 weeks	1011 (24.7%)	74 (25.0%)	40 (22.0%)
Birth weight (median grams, IQR)	961 (760–1180)	1010 (780–1218)	1030 (782–1240)
Male sex (N, %)	2271 (55.5)	156 (52.7)	102 (56.0)
Multiple births (N, %)	1004 (24.5)	78 (26.4)	40 (22.0)
**Socio-demographic characteristics of parents**			
Number of mothers (N)‡	3573	254	162
Maternal age (years, mean, SD)	30.5 (6.1)	29.5 (5.9)	28.7 (6.0)
Mothers reporting white British ethnicity (N, %)	1793 (50.2)	155 (61.0)	116 (71.6)
Non-white British	1186 (33.2)	34 (13.4)	15 (9.3)
Missing data	594 (16.6)	65 (25.6)	31 (19.1)
Level of deprivation (Index of Multiple Deprivation (IMD) quintile, N, %)§			
1 (Most deprived)	1215 (34.0)	88 (34.7)	51 (31.5)
2	804 (22.5)	60 (23.6)	30 (18.5)
3	611 (17.1)	33 (13.0)	37 (22.8)
4	487 (13.6)	36 (14.2)	20 (12.4)
5 (Least deprived)	391 (10.9)	28 (11.0)	21 (13.0)
Missing data	65 (1.8)	9 (3.5)	3 (1.9)
Any reported smoking history (N, %)	602 (16.9)	38 (15.0)	43 (26.5)
**Clinical characteristics of mothers**			
Hypertension in pregnancy (N, %)	163 (4.6)	16 (6.3)	5 (3.1)
Premature rupture of membranes (N, %)	603 (16.9)	49 (19.3)	30 (18.5)
Caesarean section (N, %)	1888 (52.8)	152 (59.8)	87 (53.7)
Antenatal steroids given (N, %)	3268 (91.5)	231 (90.9)	149 (92.0)
**Maternity Unit characteristics**			
Total number of maternity units	150	18	12
With no neonatal service	3	3	2
With special care baby unit/local neonatal unit	106	6	7
With neonatal intensive care unit	41	9	3
Births per level of unit (N, %)			
With no neonatal service	6 (0.2)	6 (2.4)	11 (6.8)
With special care baby unit/local neonatal unit	1329 (37.2)	36 (14.2)	49 (30.3)
With neonatal intensive care unit	2238 (62.6)	212 (83.5)	102 (63.0)
Average number of eligible births per hospital per month (mean, SD)	2.8 (2.1)	2.3 (1.3)	2.1 (1.6)

* Baseline period is means/proportions across January–December 2017, the year before the National PReCePT Programme (NPP) was rolled out in England.

† Babies up to 30 weeks’ gestational age. Descriptive data is on all babies including multiples. Analysis is restricted to singletons and first-born of multiples.

‡ Unique mother IDs.

§ English, Scottish and Welsh Indices of Multiple Deprivation are calculated differently and are not comparable between nations.

PReCePT, PRevention of Cerebral palsy in PreTerm labour.

### Historical trends

**Figure 1:**
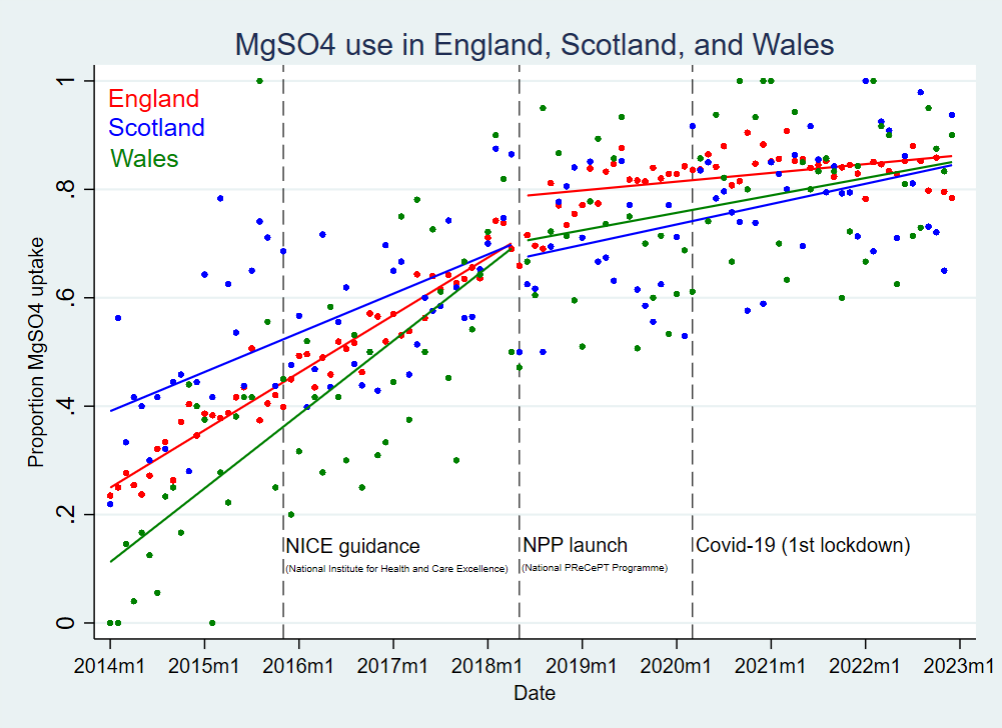
Magnesium sulfate (MgSO_4_) uptake in England, Scotland and Wales, 2014–2022. PReCePT, PRevention of Cerebral palsy in PreTerm labour.

In 2014, MgSO_4_ uptake was around 20% in England, 40% in Scotland and 10% in Wales. Uptake improved over time in all three nations, with the rate of change slowing down at higher levels of treatment (ceiling effect). Although national levels appeared to converge in the latest 2022 data, there was a visual suggestion that since the launch of the NPP, uptake may have been accelerated in England compared with the devolved nations. Due to relatively smaller numbers, there was high variation in monthly uptake for Scotland and Wales, which limited formal assessment of parallel trends (figure 1).

### Pre/post-NPP comparison

In England, overall MgSO_4_ uptake rose from 65.8% in 2017 to 85.5% in 2022 (62.3% to 81.4% in Scotland, 61.6% to 86.6% in Wales). The amount of missing data fell from around 5% in 2017 to under 1% in 2022. ‘Imminent delivery’ (a matter of clinical judgement that there was insufficient time, between presentation and delivery, to administer MgSO_4_) was the most commonly recorded reason for not giving MgSO_4_, accounting for around 15% of eligible babies in 2017, dropping to around 10% in England and Wales in 2022. The number recorded as not offered MgSO_4_ fell from around 7% to around 1% across all three nations (online supplemental table 3).

### Estimate of NPP effectiveness

The adjusted model estimated an average 5.8 (95% CI 2.7 to 8.9, p<0.001) percentage point increase in MgSO_4_ uptake in England across the 4 years post-NPP, compared with the 1-year pre-NPP. Much of the gains appeared to take place as a step change in the first 2 years of the programme. There were additional gains in years three and four (at which point the improvement became statistically significant), although CIs overlap with estimates from the first 2 years (table 2).

**Table 2:** Change in magnesium sulfate (MgSO_4_) uptake in England from before to after the National PReCePT Programme (NPP)*

	Step-change in magnesium sulfate (MgSO_4_) uptake after intervention (percentage points)†	95% CI	P value	Change in slope postintervention compared with pre-intervention	95% CI	P value	Overall slope postintervention	95% CI	P value
Unadjusted‡	11.8	10.0 to 13.6	<0.001	–	–	–			
Fully adjusted§	5.8	2.7 to 8.9	<0.001	−0.87	−1.18 to −0.57	<0.001	0.02	−0.06 to 0.10	0.622
Cumulative increase per-year across the follow-up period‡									
Year 1	3.1	−1.3 to 7.4	0.167	−0.76	−1.48 to −0.05	0.037	0.19	−0.31 to 0.69	0.450
Year 1–2	3.5	−0.2 to 7.3	0.067	−0.57	−0.91 to −0.23	<0.001	0.25	0.09 to 0.41	0.002
Year 1–3	5.6	2.1 to 9.1	0.002	−0.86	−1.25 to −0.48	<0.001	0.05	−0.05 to 0.16	0.302
Year 1–4	5.8	2.7 to 8.9	<0.001	−0.87	−1.17 to −0.57	<0.001	0.02	−0.06 to 0.10	0.622
Secondary, sensitivity and subgroup analyses‡									
Analysis at maternity unit level¶	5.8	2.8 to 8.8	<0.001	−0.83	−1.47 to −0.18	0.012	0.003	−0.05 to 0.06	0.923
Analysis at individual level¶	5.9	2.5 to 9.3	0.001	−0.76	−1.17 to −0.34	<0.001	0.02	−0.03 to 0.07	0.449
Excluding implementation start window (2 months each side of start)	6.6	2.9 to 10.3	<0.001	−0.86	−1.17 to −0.54	<0.001	−0.01	−0.09 to 0.07	0.820
Excluding final 2 months (data quality)	5.8	2.6 to 8.9	<0.001	−0.82	−1.14 to −0.50	<0.001	0.04	−0.04 to 0.12	0.346
Comparing 4 years pre with 4 years post	6.0	3.7 to 8.4	<0.001	−0.74	−0.82 to −0.65	<0.001	0.01	−0.07 to 0.08	0.895
PReCePT cluster randomised controlled trial units only (n=40)	8.6	2.4 to 14.9	0.007	−0.79	−1.37 to −0.20	<0.001	0.04	−0.09 to 0.16	0.563
Including babies up to 34 weeks gestation	8.7	6.4 to 11.0	<0.001	−0.69	−0.90 to −0.48	<0.001	0.046	−0.02 to 0.11	0.157

* All data on singletons and first born of multiples <30 weeks’ gestation and admitted to an English National Health Service (NHS) Neonatal unit.

† Percentage point difference in uptake between mean across the 12-month pre-National PReCePT Programme (NPP), and mean across the 4 years post-NPP.

‡ Crude regression of uptake postimplementation compared with preimplementation.

§ Fully adjusted model: includes interaction between pre–post period and study month to capture change in slope as well as step-change. Adjusted for covariates as monthly aggregates: mean maternal age, proportion who identified as of white British ethnicity, mean Index of Multiple Deprivation (IMD) decile, proportion of multiple births, proportion with pregnancy hypertension, reported smokers, type of birth (c-section vs vaginal delivery), birth weight adjusted for gestational age as a z-score. Model weighted on unit size.

¶ Additionally adjusted for level of birth unit and regional clustering by Academic Health Sciences network (AHSN).

PReCePT, Prevention of cerebral palsy in preterm labour.

Estimates were robust to sensitivity analyses (table 2). There was an indication of greater improvement in MgSO_4_ use when including babies up to 34 weeks’ gestational age in the analysis (8.7 percentage point increase in MgSO_4_ uptake, 95% CI 6.4 to 11.0, p<0.001) and in the 40 units in the PReCePT RCT (8.6 percentage point increase in MgSO_4_ uptake, 95% CI 2.4 to 14.9, p=0.007). There was some evidence that lower-level units improved more than higher-level units (SCBUs and LNUs: 9.1 percentage points change, 95% CI 3.9 to 14.3, p=0.001. NICUs: 4.1 percentage points change, 95% CI 0.6 to 7.6, p=0.022), reflecting the fact that NICUs tended to have higher starting levels.

### Impact of the COVID-19 pandemic

In 2020, there was a slight declining trend in MgSO_4_ use coinciding with the start of the COVID-19 pandemic, which continued to the end of the dataset at the end of 2022. The use of antenatal steroids (another, more well-established protective treatment for preterm babies) had an almost identical decline over this same period (online supplemental figure 1).

#### Economic evaluation

**Figure 2:**
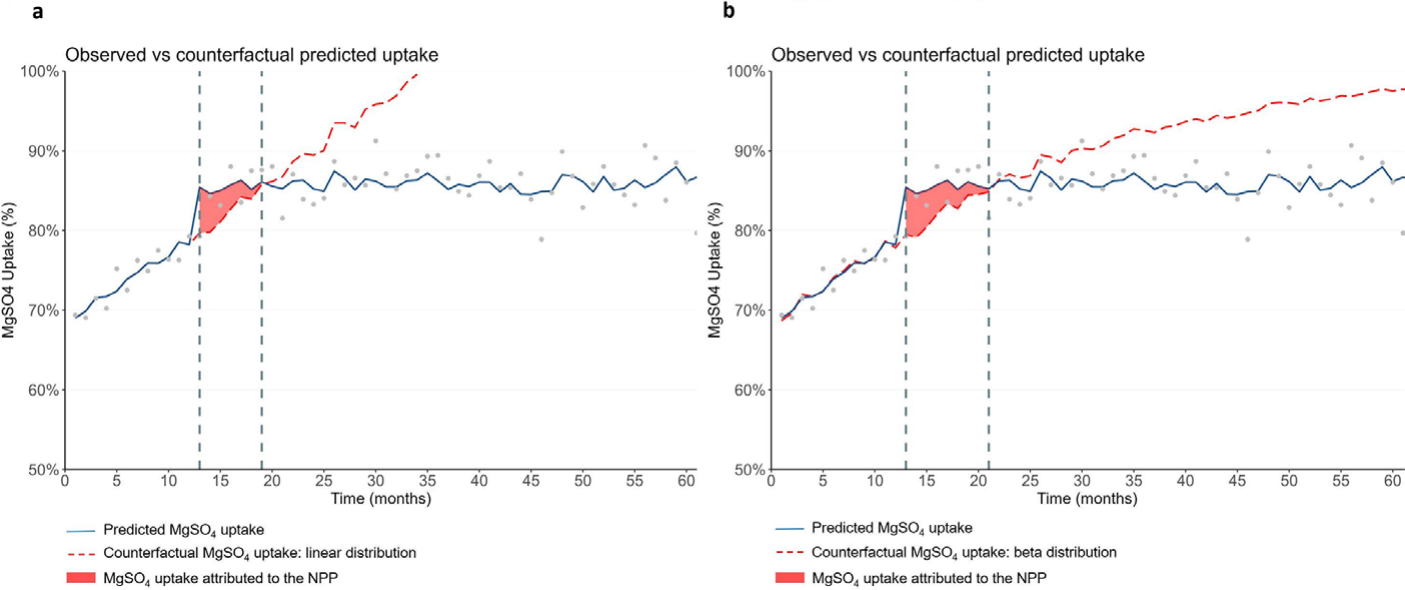
Predicted magnesium sulfate (MgSO_4_) uptake, counterfactual and area between curves from interrupted time series analyses.

The impact of the NPP is illustrated in figure 2, using a linear regression (figure 2a) and a beta regression (figure 2b) for the counterfactuals. Probabilistic analysis estimated that the additional use of MgSO_4_ attributed to the NPP was equivalent to a 3.0 percentage point improvement on average over 7 months, which equates to an additional 64 of the 2136 preterm (<30 weeks’ gestation) babies receiving treatment (table 3). The lifetime and societal NMB of the NPP was about £597 000 or £279 per preterm baby. The probability of the NPP being cost-effective was 89% (table 3, online supplemental figure 2).

**Table 3:** Probabilistic cost-effectiveness results of the National PReCePT Programme (NPP) from Interrupted Time Series Analysis (<30 weeks’ gestation)

	Main analysis (linear counterfactual)	Sensitivity analysis (beta counterfactual)
Period of benefit, months	7	10
Number of preterm babies (<30 weeks), N	2136	3129
Change in percentage of preterm babies treated with magnesium sulfate (MgSO_4_) (Δb_i_) %	3.0 (1.5; 4.5)	2.9 (2.3; 3.6)
Net Increment of preterm babies treated with MgSO_4_ (Δpat)	64 (32; 97)	92 (72; 112)
Net cost of implementation (ΔC_i_) £	936 747	936 747
Implementation cost-effectiveness (ΔCi/ΔPat) £ per additional patient treated	14 576 (29 284; 9669)	10 219 (13 040; 8386)
Lifetime health effect of MgSO_4_ treatment per patient (Δbt) (quality adjusted life years (QALY))	0.24 (0.16; 0.33)	0.24 (0.16; 0.33)
Lifetime costs of MgSO_4_ treatment per patient (Δct) £	−19 064 (−13 310; −25 648)	−19 064 (−13 310; −25 648)
Net Monetary Benefit of the Policy (NMB_P_), £*	596 538 (-221 748; 1 541 786)	1 251 511 (558 115; 2 071 244)
Probability of being cost-effective, %	89	100

* At a willingness-to-pay threshold of £20 000 per QALY gained.

PReCePT, PRevention of Cerebral palsy in PreTerm labour.

The sensitivity analysis with the beta distribution counterfactual estimated a longer period of impact (an additional 3 months) over which there was additional use of MgSO_4_ attributed to the NPP equivalent to a 2.9 percentage point improvement on average over 10 months, which equates to an additional 92 of 3129 preterm babies receiving treatment (figure 2b). Accounting for the total cost of the NPP, and the lifetime health gains and cost savings of MgSO_4_ treatment, the NMB was estimated to be £1.3 m, or £400 per preterm baby. The probability of the NPP being cost-effective in this analysis was 100% (table 3).

Expanding the analysis to include babies up to 32 weeks’ gestation estimated additional use of MgSO_4_ attributed to the NPP equivalent to a 4.4 percentage point improvement on average over 9 months, which equates to an additional 215 preterm babies treated. As the total cost of the NPP was fixed and not sensitive to the number of babies treated, the NMB of the NPP was about £4.2 m or £853 per preterm baby. The probability of NPP being cost-effective was 100% at the WTP threshold of £20 000 per QALY gained (online supplemental table 4 and online supplemental figure 3). For the sensitivity analysis with the beta distribution, the NMB of NPP was about £5.4 m, or £700 per preterm baby. The probability of cost-effectiveness was 100% (online supplemental table 4).

Expanding the analysis further again to include babies up to 34 weeks’ gestation shows that the additional use of MgSO_4_ attributed to the NPP was equivalent to a 7.3 percentage point improvement on average over 7 months. This means an additional 961 babies receiving treatment. Using a beta distribution for the counterfactual showed a 6.5 percentage point improvement attributed to the NPP over 12 months (698 additional patients). The NMB of the NPP was not calculated for this more mature group of preterm babies, as currently there is no available estimate for the cost-effectiveness of MgSO_4_ treatment for babies born above 31^+6^ weeks’ gestation.

## Discussion

The original NPP evaluation found evidence of improved MgSO_4_ use over the first 12 months following implementation.^12^ This extended evaluation found that the improvements have largely been sustained over the first 4 years following implementation, although there was suggestion of a slight decline in use coinciding with the pandemic. The benefits applied both to the target population of births <30 weeks’ gestation, but also to more mature preterm babies up to 34 weeks’ gestation. The programme was associated with an NMB of about £0.6 m for babies up to 30 weeks’ gestation, rising to about £4.2 m when babies up to 32 weeks’ gestation are included. Compared with the devolved nations, uptake appeared to improve faster in England in the first 2 years following the NPP launch. By the end of 2022, however, the three nations were broadly comparable with the delivery of MgSO_4_ to around 81–87% of eligible mothers. This is at the higher end of levels reported internationally (69–87%^29^,^32^) following guidelines or interventions to increase MgSO_4_ uptake.

Comparisons between England, Scotland and Wales should be interpreted with caution: first, because there is high variability due to small numbers in the devolved nations data, which limits formal statistical comparison of trends; second, because Scotland and Wales were implementing their own MgSO_4_ initiatives (eg, MCQIC Preterm Perinatal Wellbeing Package,^14^ PERIPrem Cymru^15^), complicating their position as a control group; third, because they were also accessing the English PReCePT toolkit and implementation resources during this time period (66 downloads from Wales, 32 from Scotland, 2018–2022, AHSN data, unpublished)—this ‘contamination’ means that the boundaries of the target population are fuzzy, and again the devolved nations cannot be considered optimal controls; finally, the three nations’ trends in uptake prior to the NPP were also not parallel, due to variation in starting levels in 2014, and this (together with the ceiling effect on uptake) meant that formal statistical comparison of their improvements was not appropriate.

The COVID-19 pandemic could plausibly have impacted on MgSO_4_ use via staffing pressures affecting all parts of the NHS,^33^ and a specific impact on expecting mothers who may have presented at hospital later due to concerns about infection and giving birth alone, both leading to missed treatment opportunities.^34 35^ Analysis of future data will be important to understand more about the observed postpandemic decline in use of both MgSO_4_ and antenatal steroids. It is likely to be more difficult to improve from 85% to 90% uptake, compared with improving from 65% to 70% uptake. Further overall increases in MgSO_4_ use may be a challenge without concerted effort at the lower-performing units. However, as some units do report higher (>90%) uptake (perhaps through better triaging and monitoring of symptoms), arguably their performance should be used as the benchmark for quality of care.

The creation of clinical guidelines alone is often not enough to ensure that evidence-based interventions become standard practice. A relevant example here is the case of antenatal steroids, which in the absence of a programme dedicated to getting this evidence into practice, took several decades for their use to become standard care. In contrast, and in the context of the NPP, the same improvements in use of MgSO_4_ were achieved within a few years.

### Strengths and limitations

This evaluation included effectiveness and cost-effectiveness analysis to capture the impact of the NPP implementation. These have been highlighted as key components of implementation science research.^20 36^ Results from the main and sensitivity analyses were consistent. Data covered a period of 8 years, giving adequate time for analysis of trends. The study benefitted from high-quality, routinely collected, national, longitudinal patient-level data. Key advantages of these comprehensive real-world data are that they provide high generalisability (including all maternity units in England, Scotland and Wales, reflecting the nationwide situation), show effectiveness in real-world conditions, and are less vulnerable to some biases such as recall, observer and attrition bias. A limitation is that these data do not necessarily include all the covariates of interest, and data quality and completeness are not always consistent across all sites. Economic evaluation has the strength of combining the evidence of the implementation (NPP) with the evidence of the treatment (MgSO_4_) and using the WTP lower threshold from NICE (£20 000 per QALY). Higher WTP thresholds, like those based on gross domestic product per capita (eg, WHO-CHOICE),^37^ would result in the NPP being more cost-effective.

A key limitation is that residual confounding cannot be excluded. We have tried to minimise the impact of confounding through robust analytic methods, and interpret findings with caution. In addition to the PReCePT programme, other factors likely impacted uptake, including: the publication of definitive evidence on the protective effect of MgSO_4_ in 2009;^38^ MgSO_4_ use being reliably recorded as a Neonatal Data Analysis Unit audit quality metric for maternity units in 2014–2015; and its use becoming a formal recommendation in the NICE Guidance in 2015.^39^ Other factors related to PReCePT include the original pilot study publishing positive results in 2017; and discussions with unit leads about the proposed NPP in 2017. Residual confounding is hard to eliminate in observational and quasi-experimental study designs, and ideally, public health interventions would be initially rolled out as a randomised controlled or stepped-wedge trial. In practice, this rarely happens, due to political and administrative timelines, practicalities and funding limits. We have tried to minimise the risk of confounding and indicated caution where appropriate in the interpretation of results.

Another limitation is that the population investigated here included liveborn babies admitted to a neonatal unit, rather than the total population of mothers eligible for MgSO_4_. The rate of MgSO_4_ uptake may be different between these two populations, although any health benefit would only be realised in those investigated in this work. It would be an advantage if future research could explore uptake and outcomes in both populations.

Finally, the economic evaluation may underestimate MgSO_4_’s economic impact on CP due to limited evidence on CP’s lifetime consequences and its extrapolation to the UK context.^40^ Bickford and colleagues’ work^6^ provided the best available estimate for this study. However, more updated and UK-specific estimates would highlight CP’s substantial economic implications and the value of prioritising strategies to reduce CP.

The Health Foundation and Health Data Research UK recently listed PReCePT as a case study model for a Learning Health System (ie, a systematic approach to iterative, data-driven QI^41^) and we propose that the PReCePT model could be used as an implementation blueprint for other QI initiatives.

## Conclusions

Implementation of the NPP has plausibly helped accelerate uptake of MgSO_4_ in England, improving maternal and neonatal care and positively impacting society in terms of direct patient benefit and future cost savings. Failure to deliver MgSO_4_ to eligible mothers should be considered inadequate care and not financially sustainable for the NHS. MgSO_4_ as a quality metric should continue to be closely monitored, and further intervention may be warranted to achieve optimal treatment levels. Future research should quantify the patient outcomes in this same population, specifically the cases of CP prevented, associated with the improvements in use of MgSO_4_.

## Supplementary material

10.1136/bmjqs-2024-017763 online supplemental file 1

10.1136/bmjqs-2024-017763 online supplemental file 2

## Data Availability

Data may be obtained from a third party and are not publicly available.
