# Supplementary material for: Implementation of national guidelines on antenatal magnesium sulfate for neonatal neuroprotection: extended evaluation of the effectiveness and cost-effectiveness of the National PReCePT Programme in England
Source: BMJ Qual Saf. 2025 Apr 27;35(2):e017763. doi: 10.1136/bmjqs-2024-017763 (PMC12911564; doi:10.1136/bmjqs-2024-017763)
Supplement: online supplemental file 1 [file bmjqs-35-2-s001.docx]

**Supplementary file**

**Supplementary Table 1: Estimated lifetime costs and QALYs per patient associated with Magnesium Sulfate (MgSO4) treatment (2019 prices)**

| **Type of birth** | **Perspective** | **Received Magnesium Sulphate (MgSO_4_)** | **Cost, £** | **Δcost, £** | **Quality Adjusted Life Years (QALYs)** | **ΔQALYs** |  |
| --- | --- | --- | --- | --- | --- | --- | --- |
| Imminent | Societal | Yes | 61971 | -23690 | 26.6 | 0.3 | |
|  |  | No | 85661 |  | 26.3 |  |  |
| Threatened | Societal | Yes | 44068 | -15964 | 26.7 | 0.2 | |
|  |  | No | 60032 |  | 26.5 |  |  |
| **Combined (40% imminent)** | **Societal** | **Yes** | **51229** | **-19054** | **26.7** | **0.24** | |
|  |  | **No** | **70284** |  | **26.4** |  |  |

Based on Bickford et al. 2013

Cost estimates were converted to Pounds Sterling and inflated to 2019 prices. First, we accounted for inflation using a Gross Domestic Product deflator index. Second, we adjusted to pound using a conversion factor from Purchasing Power Parities values. Both retrieved from Internation Monetary Fund – World Economic Outlook Database (https://www.imf.org/en/Publications/SPROLLs/world-economic-outlook-databases#sort=%40imfdate%20descending)

**Supplementary table 2: Point estimates, probability distributions, and source of parameter estimates used in the probabilistic analysis**

| **Analysis** | **Element** | **Statistics** | **Linear** | **Distribution** | **Source** |  |
| --- | --- | --- | --- | --- | --- | --- |
| **Common estimates** | Health utility, Quality Adjusted Life Years (QALYs) | Mean (Standard Error (SE)*) | 0.24 (0.05) | Beta distribution | Bickford et al. | |
|  | Lifetime costs, £ | Mean (SE *) | -19054 (-3811) | Gamma distribution | Bickford et al. | |
|  | Cost National PReCePT Programme (NPP), £ | Total | 936747 | N/A | PReCePT Study – Cost analysis | |
| **Linear Interrupted Time Series (ITS) <30 weeks gestation** | Pre-term babies, *N* | Sum | 2136 | N/A | NNRD data | |
|  | Change in the proportion of Magnesium Sulphate (MgSO_4_) | Mean (SE) | 3.0% (0.9%) | Normal distribution | Area-Between-Curves | |
| **Beta ITS <30 weeks gestation** | Pre-term babies, *N* | Sum | 3129 | N/A | NNRD data | |
|  | Change in the proportion of MgSO_4_ | Mean (SE) | 3.0% (0.4%) | Normal distribution | Area-Between-Curves | |
| **Linear ITS <32 weeks gestation** | Pre-term babies, *N* | Sum | 4923 | N/A | NNRD data | |
|  | Change in the proportion of MgSO_4_ | Mean (SE) | 3.4% (1.0%) | Normal distribution | Area-Between-Curves | |
| **Beta ITS <32 weeks gestation** | Pre-term babies, *N* | Sum | 7768 | N/A | NNRD data | |
|  | Change in the proportion of MgSO_4_ | Mean (SE) | 3.4% (0.3%) | Normal distribution | Area-Between-Curves | |

*Standard errors are calculated as the 20% of the point estimates as in Bickford et al. 2013

**Supplementary table 3: Magnesium Sulfate (MgSO_4_) uptake in England, Scotland, and Wales, pre- and post-National PReCePT Programme (NPP)^0^**

|  | **England** | | **Scotland** | | **Wales** | |
| --- | --- | --- | --- | --- | --- | --- |
|  | **2017**^1^ | **2022**^2^ | **2017**^1^ | **2022**^2^ | **2017**^1^ | **2022**^2^ |
| Total number of eligible births^0^ | 3573 | 3286 | 254 | 253 | 162 | 135 |
| Total number of mothers **given** MgSO_4_ (%) | 2223  (62.2%) | 2786  (84.8%) | 149  (58.7%) | 205  (81.0%) | 93  (57.4%) | 116  (85.9%) |
| Total number of mothers **not** **given** MgSO_4_ (%) | 1158  (32.4%) | 474  (14.4%) | 90  (35.6%) | 47  (18.6%) | 58  (35.8%) | 18  (13.3%) |
| Total number with **missing** MgSO_4_ data (%) | 192  (5.4%) | 26  (0.8%) | 15  (5.9%) | 1  (0.4%) | 11  (6.8%) | 1  (0.7%) |
| **MgSO_4_ uptake^3^** (sd) | 65.8%  (0.5) | 85.5%  (0.4) | 62.3%  (0.5) | 81.4%  (0.4) | 61.6%  (0.5) | 86.6%  (0.3) |
| Reason MgSO_4_ not given (%)^4^ |  |  |  |  |  |  |
| Contraindicated | 11 (0.3%) | 5 (0.2%) | 2 (0.8%) | 1 (0.4%) | 0 (0.0%) | 1 (0.7%) |
| Declined | 3 (0.1%) | 3 (0.1%) | 0 (0.0%) | 1 (0.4%) | 1 (0.6%) | 0 (0.0%) |
| Delivery imminent | 548 (15.3%) | 329 (10.0%) | 42 (16.5%) | 38 (15.0%) | 23 (14.2%) | 13 (9.6%) |
| Not appropriate | 126 (3.5%) | 23 (0.7%) | 14 (5.5%) | 1 (0.4%) | 12 (7.4%) | 0 (0.0%) |
| Not offered | 240 (6.7%) | 32 (1.0%) | 18 (7.1%) | 2 (0.8%) | 14 (8.6%) | 2 (1.5%) |
| Data missing | 230 (6.4%) | 82 (2.5%) | 14 (5.5%) | 4 (1.6%) | 8 (4.9%) | 2 (1.5%) |

0. All data on singletons and first born of multiples <30 weeks’ gestation and admitted to an NHS Neonatal unit

1. Across the year Jan-Dec 2017

2. Across the year Jan-Dec 2022

3. Uptake percentage calculated excluding missing values from the denominator, to fit with national audit reporting practices.

4. Percentage calculated out of total cases

**Supplementary table 4: Probabilistic Cost-Effectiveness Results of the National PReCePT Programme (NPP) from Interrupted Time Series (ITS) Analysis (<32 weeks’ gestation)**

|  | **Main analysis**  (linear counterfactual) | **Sensitivity analysis**  (beta counterfactual) |
| --- | --- | --- |
| Period of benefit, months | 9 | 12 |
| Number of pre-term babies (<32 weeks), N | 4923 | 7768 |
| Change in percentage of pre-term babies treated with Magnesium Sulphate (MgSO_4_) (Δb_i_) % | 4.4% (2.7%; 6.0%) | 3.4% (3.0%; 3.9%) |
| Net Increment of pre-term babies treated with MgSO_4_ (Δpat) | 215 (133; 297) | 267 (230; 303) |
| Net cost of implementation (ΔC_i_) £ | 936747 | 936747 |
| Implementation cost-effectiveness (ΔCi / ΔPat) £ per additional patient treated | 4350 (7031; 3153) | 3508 (4065; 3087) |
| Lifetime health effect of MgSO_4_ treatment per patient (Δbt) Quality Adjusted Life Years (QALYs) | 0.24 (0.16; 0.33) | 0.24 (0.16; 0.33) |
| Lifetime costs of MgSO_4_ treatment per patient (Δct) £ | -19064 (-13310; -25648) | -19064 (-13310; -25648) |
| Net Monetary Benefit of the Policy (NMB_P_), £^1^ | 4199799 (1986016; 6803391) | 5433488 (3668040; 7508699) |
| Probability of being cost-effective, % | 100% | 100% |

^1^ At a willingness-to-pay threshold of £20,000 per QALY gained

**Supplementary Figure 1:**

**
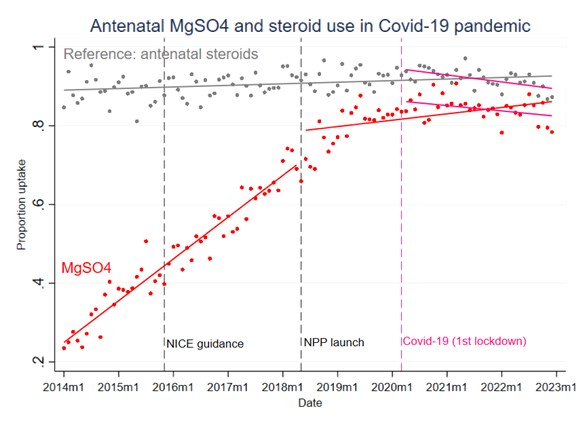
**

NICE: The UK National Institute for Health and Care Excellence

NPP: National PReCePT Programme

MgSO_4_: Magnesium Sulfphate

**Supplementary Figure 2: Cost-Effectiveness Plane and Cost-Effectiveness Acceptability Curve from Linear Interrupted Time Series analysis (<30 weeks gestation)**


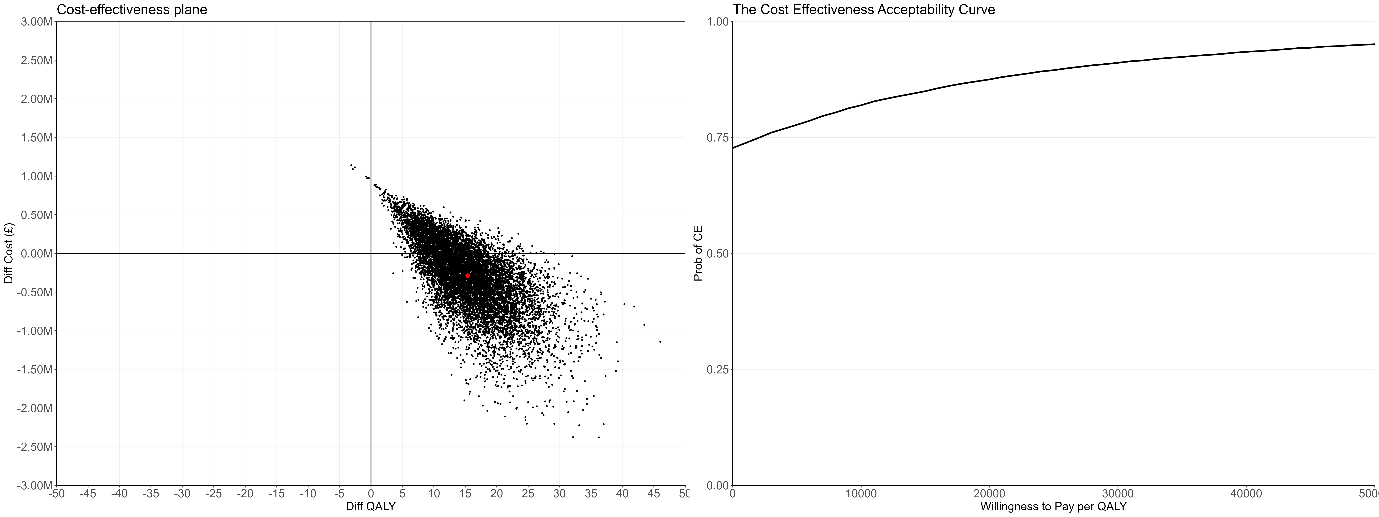


The graph on the left displays the results of Monte Carlo simulations with 10,000 iterations using the value ranges and distributions presented in Appendix 2. The horizontal axis represents the effect measures in Quality Adjusted Life Years (QALYs) for the National PReCePT Programme, and the vertical axis represents the cost. Datapoints falling in the top right quadrant indicate that the National PReCePT Programme was effective and costly. Datapoints falling bottom right quadrant indicate that the National PReCePT Programme was effective and cost-saving.

**Supplementary Figure 3: Cost-Effectiveness Plane and Cost-Effectiveness Acceptability Curve from Linear Interrupted Time Series analysis (<32 weeks gestation)**


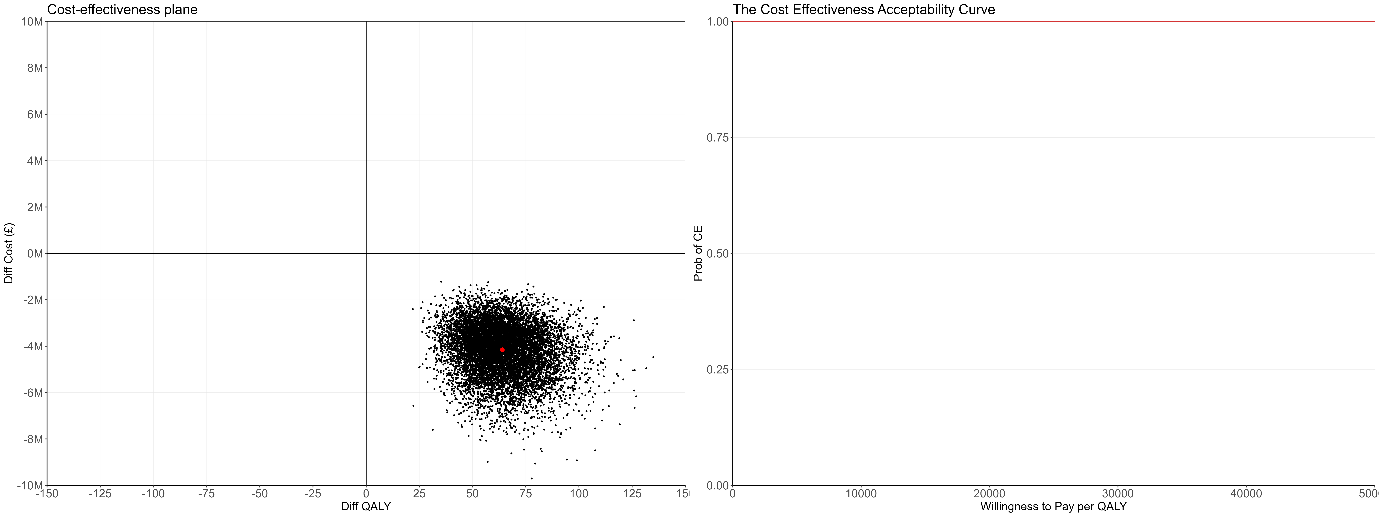


The graph on the left displays the results of Monte Carlo simulations with 10,000 iterations using the value ranges and distributions presented in Appendix 2. The horizontal axis represents the effect measures in Quality Adjusted Life Years (QALYs) for the National PReCePT Programme (NPP), and the vertical axis represents the cost. Datapoints falling bottom right quadrant indicate that the National PReCePT Programme (NPP) was effective and cost-saving.
